# Catechol biosynthesis from glucose in *Escherichia coli* anthranilate-overproducer strains by heterologous expression of anthranilate 1,2-dioxygenase from *Pseudomonas aeruginosa* PAO1

**DOI:** 10.1186/s12934-014-0136-x

**Published:** 2014-10-04

**Authors:** Víctor E Balderas-Hernández, Luis G Treviño-Quintanilla, Georgina Hernández-Chávez, Alfredo Martinez, Francisco Bolívar, Guillermo Gosset

**Affiliations:** Departamento de Ingeniería Celular y Biocatálisis, Instituto de Biotecnología, Universidad Nacional Autónoma de México, Apdo, Postal 510-3, Cuernavaca, Morelos CP 62271 Mexico; Laboratorio de Biología Integrativa de Plantas y Microorganismos, Unidad Académica de Ciencias Biológicas, Universidad Autónoma de Zacatecas, Av. Preparatoria s/n, Col., Agronómica, CP 98066 Zacatecas Mexico; Departamento de Tecnología Ambiental, Universidad Politécnica del Estado de Morelos, Jiutepec, Morelos Mexico

**Keywords:** Aromatics, Catechol, Anthranilate, Metabolic engineering, *Escherichia coli*, Anthranilate 1,2-dioxygenase

## Abstract

**Background:**

The aromatic compound catechol is used as a precursor of chemical products having multiple applications. This compound is currently manufactured by chemical synthesis from petroleum-derived raw materials. The capacity to produce catechol is naturally present in several microbial species. This knowledge has been applied to the generation of recombinant *Escherichia coli* strains that can produce catechol from simple carbon sources.

**Results:**

Several strains derived from *E. coli* W3110 *trpD9923*, a mutant that overproduces anthranilate, were modified by transforming them with an expression plasmid carrying genes encoding anthranilate 1,2-dioxygenase from *Pseudomonas aeruginosa* PAO1. The additional expression of genes encoding a feedback inhibition resistant version of 3-deoxy-D-*arabino*-heptulosonate 7-phosphate (DAHP) synthase and transketolase from *E. coli*, was also evaluated. Generated strains were characterized in complex or minimal medium in shake-flask and fed-batch bioreactor cultures and incubation temperatures ranging from 37 to 28°C. These experiments enabled the identification of culture conditions for the production of 4.47 g/L of catechol with strain W3110 *trpD9923*, expressing 1,2-dioxygenase, DAHP synthase and transketolase. When considering the amount of glucose consumed, a yield of 16% was calculated, corresponding to 42% of the theoretical maximum as determined by elementary node flux analysis.

**Conclusions:**

This work demonstrates the feasibility of applying metabolic engineering for generating *E. coli* strains for the production of catechol from glucose via anthranilate. These results are a starting point to further optimize environmentally-compatible production capacity for catechol and derived compounds.

## Introduction

Aromatic compounds are used in industry mainly as building blocks for the generation of various valuable products. The synthesis of aromatics using metabolic engineering and synthetic biology is an arising and promising alternative to the conventional chemical production methods. The biotechnological production of aromatic compounds relies mainly on the use of microorganisms that naturally synthesize a broad range of organic compounds, and more importantly, on the utilization of renewable starting materials different from petroleum derivatives. The production of catechol is among one of the multiple examples of how biotechnology is changing traditional chemical synthesis methods. Catechol (1,2-dihydroxybenzene) is an aromatic compound used in a variety of applications. It is employed as a reagent for photography, dyestuffs, electroplating, rubber and plastics production, also as starting material for the production of insecticides, perfumes and some drugs [1]. The oxidation of phenol and *m*-diisopropylbenzene and the distillation of coal-tar are the main methods for catechol production [1]. However, these procedures are expensive, lengthy and non-environmentally sustainable, due to their characteristics in terms of reactant requirements and reaction conditions. Alternatively, several *Pseudomonas* species display the capacity of growing utilizing aromatic hydrocarbons, transforming them into catechol or protocatechuate. This metabolic capacity has been employed for producing catechol using microorganisms as biocatalysts [2]. In a *Pseudomonas putida* strain expressing the enzyme toluene/benzene dioxygenase and lacking catechol 1,2-oxygenase and catechol 2,3-oxygenase activities, catechol was produced by benzene oxidation [3]. Also in a *Bacillus stearothermophilus*, strain BR219, capable of degrading phenol, the addition of tetracycline during the stationary phase resulted in the inactivation of the plasmid encoded catechol 2,3-dioxygenase from the phenol meta pathway of this microorganism, resulting in the accumulation of catechol [4]. Alternative to this strategy, transposon Tn916 was used to disable the catechol 2,3-dioxygenase gene in *B. stearothermophilus* [5].

The first example of *Escherichia coli* strains designed for catechol production was based on genetic modifications to cause accumulation of the intermediate 3-dehydroshikimic acid (DHS) and expression of genes encoding heterologous DHS dehydratase and protocatechuic acid decarboxylase. One of these recombinant *E. coli* strains produced catechol with a 33% yield from glucose [6]. In another example, a 2% increase in yield of catechol was observed when applying a resin-based extraction process to reduce the toxic effect of this product [7]. In these strains, the genetic modification causing DHS accumulation also results in a multiple auxotrophy for aromatic amino acids and vitamins, therefore, culture medium supplementation is required.

**Figure 1:**
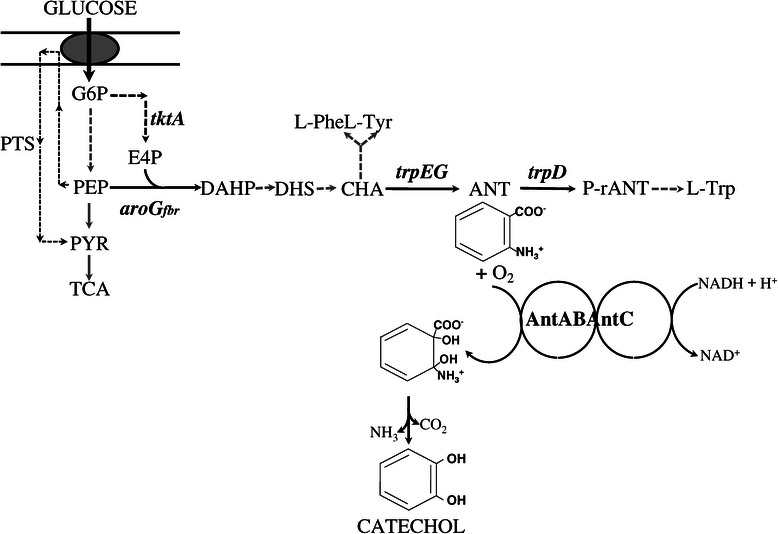
**Metabolic network related to catechol biosynthesis in** ***E. coli*** **.** Arrows with dashed lines indicate more than one enzymatic reaction. Metabolite symbols: G6P, glucose 6-phosphate; PYR, pyruvate; PEP, phosphoenolpyruvate; E4P, erythrose 4-phosphate; DAHP, 3-deoxy-D-*arabino*-heptulosonate 7-phosphate; CHA, chorismate; DHS, dehydroshikimic acid; ANT, anthranilate; L-Phe, L-phenylalanine; L-Tyr, L-tyrosine; L-Trp, L-tryptophan; TCA, tricarboxylic acid cycle; PTS, phosphotransferase transport system; *tktA*, transketolase; *aroG* ^fbr^, feedback inhibition resistant DAHP synthase; *trpED*, anthranilate synthase-phosphoribosyl transferase complex; AntAB, terminal oxygenase component and AntC, reductase component of anthranilate 1,2-dioxygenase.

Our group previously generated and characterized a recombinant bacterial system for the production of anthranilate from glucose based on *E. coli* strain W3110 *trpD9923*. This strain displays a low anthranilate producing capacity, resulting from a mutation in the *trpD* gene (the anthranilate phosphoribosyl transferase function) of the tryptophan operon (Figure 1). Derivatives of this strain were generated by following strategies known to increase carbon flow to the synthesis of the aromatic amino acids. The central metabolism precursors phosphoenolpyruvate (PEP) and erythrose 4-phosphate were redirected into the common aromatic pathway by transforming W3110 *trpD9923* with plasmid pJLB*aroG*^fbr^*tktA*. This plasmid carries the genes encoding a feedback inhibition resistant version of the enzyme 3-deoxy-D-*arabino*-heptulosonate-7-phosphate (DAHP) synthase (*aroG*^fbr^) and transketolase (*tktA*). To avoid consumption of PEP during glucose import, the phosphoenolpyruvate:sugar phosphotransferase system (PTS) was inactivated (Figure 1). One of the generated strains, W3110 *trpD9923*/pJLB*aroG*^fbr^*tktA* produced 14 g/L of anthranilate in fed-batch cultivation with glucose [8].

Various microorganisms have enzymes that catalyze mono- and dioxygenation reactions of aromatic substrates such as benzene, phenol, aniline, toluene, anthranilate, among others, to generate catechol, which can be further catabolized via the β-ketoadipate pathway to generate tricarboxylic acid cycle (TCA) intermediates [9]. The strain *P. aeruginosa* PAO1 is able to use anthranilate as a carbon and energy source [10]. In this bacterium, via *ortho*-dihydroxylation followed by spontaneous deamination and decarboxylation, anthranilate is converted to catechol. The hydroxylation reaction is catalyzed by a class IB two-component anthranilate 1,2-dioxygenase (AntDO), which comprises the terminal oxygenase component (AntAB) and the reductase component (AntC) (Figure 1) [11,12].

In this work, we explored the feasibility of producing catechol from glucose via anthranilate with strains having a modification that causes a single auxotrophy for L-Trp. We genetically engineered several anthranilate-producing strains derived from W3110 *trpD9923* by expressing the *antABC* genes coding for the AntDO of *P. aeruginosa* PAO1 (Figure 1) and determined their capacity for producing catechol under various culture conditions.

The existence of two alternate approaches for microbial catechol production from simple carbon sources will allow studies to determine which one will result in strains and production processes displaying desirable characteristics such as high strain robustness and specific productivity as well as low culture medium cost. This report will provide initial data allowing such comparisons to be made.

## Material and methods

### Strains and plasmids

The *E. coli* strains and plasmids used in this study are described in Table 1. *E. coli* strain W3110 *trpD9923* was obtained from the *E. coli* Genetic Stock Center (Yale University, New Haven, CT). *E. coli* W3110 *trpD9923* strain is a mutant in the *trpD* gene of the tryptophan operon obtained by treatment with ultraviolet radiation, thus, it accumulates anthranilate and it is a tryptophan auxotroph [13]. The W3110 *trpD9923* PTS^−^ strain is a derivative of W3110 *trpD9923* with an inactivated PTS operon (Δ*ptsH*, *ptsI*, *crr*::Km^R^) [8]. Plasmid pJLB*aroG*^fbr^*tktA* carries the *aroG*^fbr^ and *tktA* genes encoding a feedback inhibition resistant version of 3-deoxy-D-*arabino*-heptulosonate 7-phosphate synthase and transketolase, respectively [14]. Plasmid pTrc-*ant3* carries the *antABC* genes coding for the anthranilate 1,2-dioxygenase (AntDO) of *P. aeruginosa* PAO1. The genes were amplified by PCR using total DNA from *P. aeruginosa* PAO1 [15] as template and the forward primer 5′-CGAGGCGT**TCTAGA**TCACCGGCGTGAC-3′ containing the *Xba*I site (in bold) and the reverse primer 5′-GTGAGAAC**CCATGG**ACGCTACCCGCAG-3′ containing the *Nco*I site (in bold). The *antABC* PCR product (2983 bp) was first cloned into pCR-Blunt II-TOPO plasmid (Invitrogen, Carlsbad, CA) and then subcloned into the pTrc99A plasmid in the sites *Xba*I and *Nco*I, under the control of the *trc* promoter.

**Table 1:** ***Escherichia coli*** **strains and plasmids used in this work**

**Strain/Plasmid**	**Characteristics**	**Reference**
Strains		
W3110 *trpD9923*	W3110 [F^−^ Δ^−^ INV (*rrn*D-*rrn*E) 1] tryptophan auxotroph, randomly mutagenized by treatment with ultraviolet radiation.	[13]
W3110 *trpD9923* PTS^−^	As W3110 *trpD9923* but Δ *ptsHI*, *crr*::km^R^, glucose^−^	[8]
Plasmids		
pJLB*aroG* ^fbr^ *tkt*A	Containing the *tktA* gene with its native promoter. *aroG* ^fbr^ expressed from the *lacUV5* promoter, *lacI* ^q^ and *tet* genes, tetracycline resistance, pACYC184 replication origin.	[8]
pTrc-*ant3*	Carries the *antABC* genes from *Pseudomonas aeruginosa* PAO1 under the control of the *trc*-derived promoter. Ampicillin resistance.	This work

### Growth media, inoculum preparation and culture conditions

Cells were routinely grown in Luria Bertani (LB) broth or LB-agar plates. Experiments in flask cultures were carried out using defined medium, containing 10 g/L glucose, 6 g/L Na_2_HPO_4_, 0.5 g/L NaCl, 3 g/L KH_2_PO_4_, 1 g/L NH_4_Cl, 246.5 mg/L MgSO_4_, 14.7 mg/L CaCl_2_ and 10 μg/mL vitamin B1, and supplemented with 20 μg/mL tryptophan. Salts, glucose, vitamin B1, MgSO_4_, tryptophan, and CaCl_2_ solutions were sterilized separately. Medium for bioreactor cultures contained 3 g/L Na_2_HPO_4_, 3 g/L KH_2_PO_4_, 1.7 g/L (NH_4_)_2_HPO_4_ and 1 mL/L of trace elements solution. This solution contains 2.7 g/L FeCl_3_, 2 g/L ZnCl_3_, 2 g/L CoCl_2_•6H_2_O, 2 g/L Na_2_MoO_4_•2H_2_O, 2 g/L CaCl_2_•2H_2_O, 0.5 g/L H_3_BO_3_ and 100 mL/L HCl. A fed-batch culture strategy was employed to provide a relatively high amount of glucose (90 g/L), divided in feeding stages to avoid osmotic shock effects. Bioreactor culture medium initially contained 30 g/L of glucose. A total of two independent pulses of 30 g/L glucose, as 25 mL of 60% sterile glucose solution, were added to the bioreactor whenever glucose concentration in the medium decreased to 10 g/L as measured off-line using an enzymatic analyzer. For some experiments, bioreactor medium was supplemented with 10 g/L of yeast extract at the beginning of the experiment and two pulses of 10 g/L yeast extract were added. Antibiotics were added to the corresponding cultures at a final concentration of 200 μg/mL ampicillin, 20 μg/mL tetracycline and 30 μg/mL kanamycin during selection, propagation and fermentation stages. Inoculum preparation was started using strain samples from frozen vials that were cultured overnight at 37°C in LB-agar plates, a single colony from these plates was used to inoculate baffled shake flasks containing mineral medium supplemented with 0.2% of glucose and 20 μg/mL tryptophan. After overnight growth at 37°C a sample was used for inoculation. For bioreactor cultures experiments, colonies from LB-agar plates were grown in 500 mL shake flasks with 100 mL mineral medium supplemented with 0.2% of glucose, 20 μg/mL tryptophan and 0.1% of peptone. Flask culture experiments were done in 250 mL flasks containing 50 mL of mineral medium, inoculated at an initial optical density at 600 nm (OD_600nm_) of 0.1 and incubated at 37°C and 300 rpm in an orbital shaker (Series 25, New Brunswick Scientific, Inc., NJ). Bioreactor cultures were performed in 1 L stirred tank bioreactors (Applikon, The Netherlands), using a working volume of 500 mL. Cultures were inoculated at an initial OD_600nm_ of 0.5, since a lower inoculum concentration resulted in a very long lag phase. pH was maintained at 7.0 by automatic addition of a 12.5% NH_4_OH solution. Airflow was set to 1 vvm. Dissolved oxygen tension was measured with a polarographic oxygen electrode (Applisens, Applikon) and maintained above 20% air saturation during all cultivation period by modifying the impeller speed. The cultivation temperature was controlled at 37, 32, or 28°C depending of the experiment as indicated. For cultures of strains carrying plasmid pJLB*aroG*^fbr^*tktA* and/or pTrc-*ant3*, gene induction was started by adding IPTG to a final concentration of 0.1 mM at an OD_600nm_ of 0.6 for shake flask and 3.0 for bioreactor cultures. Inducer IPTG was employed at a final concentration of 0.1 mM based on experiments showing similar strain performance when compared to conditions having a final concentration of 1 mM.

### Kinetic parameters calculation

For the characterization of the strains used in this work, specific rates of growth (μ), glucose consumption (*q*_Glc_), anthranilate production (*q*_Ant_) and catechol production (*q*_Cat_), as well as yields of anthranilate (Y_Ant/Glc_) and catechol (Y_Cat/Glc_) on glucose were determined. μ and *q*_Glc_ were calculated during the exponential growth phase. Since growth rates and production kinetics of catechol and anthranilate differed among studied strains, *q*_Ant_, *q*_Cat_, Y_Ant/Glc_, and Y_Cat/Glc_ were calculated considering only the catechol or anthranilate production phase, defined as the time period starting when catechol or anthranilate was detected up to the point when a sharp decrease in product accumulation was observed. Flask cultures were performed in triplicate and bioreactor cultures in duplicate. The values reported represent the mean of all performed experiments and standard errors are shown in tables.

### Analytical methods

Biomass concentration was measured as OD_600nm_ using a spectrophotometer (Beckman DU-70, Palo Alto, CA) and converted to dry cell weight (DCW) considering that 1 OD_600nm_ = 0.37 g_DCW_/L [16]. Samples taken during cultivation period were centrifuged at 10 000 rpm for 2 min. Supernatant was filtered using 0.45 μm syringe-filter and stored at −20°C for subsequent analysis. Glucose was determined using an enzymatic analyzer (YSI 2700, YSI Life Sciences, OH). Acetate was determined by high performance liquid chromatography (HPLC) (Waters, Milford, MA), using an Aminex HPX-87H column (300x7.8 mm; Bio-Rad, Hercules, CA); running conditions were 5 mM H_2_SO_4_ as mobile phase, flow of 0.5 mL/min and temperature of 50°C. Detection was performed by photodiode array at 210 nm. Catechol and anthranilate were determined from the culture medium and not from biomass, since the former is the fraction available for recovery. The method employed was HPLC (Agilent Technologies, Palo Alto, CA) using a Synergy Hydro C_18_ 4 μm column (4.6x150 mm, Phenomenex, Torrance, CA); running conditions were 0.1% trifluoroacetic acid in 40% methanol as mobile phase, flow of 0.5 mL/min. Detection was performed by photodiode array at 330 nm for anthranilate and at 280 nm for catechol. The maximum theoretical yield for synthesis of catechol or anthranilate from glucose corresponds to 0.376 g/g and was determined by applying elementary mode flux analysis using METATOOL [17].

## Results

### Production of catechol in shake-flask cultures with derivatives of *E. coli* W3110 *trpD9923*

The enzyme AntDO from *P. aeruginosa* PAO1, encoded by *antABC* genes, catalyzes the conversion of anthranilate to catechol. To generate an *E. coli* strain for the production of catechol via anthranilate, the *antABC* genes were amplified from *P. aeruginosa* PAO1 and cloned in an expression vector, thus generating plasmid pTrc-*ant3*. Based on data described by Balderas et al. (2009), strains W3110 *trpD9923*, W3110 *trpD9923* PTS^−^ and W3110 *trpD9923*/pJLB*aroG*^fbr^*tktA* were selected for this study since they displayed a wide range of anthranilate production capacity from glucose. These three strains were transformed with plasmid pTrc-*ant3.*

**Figure 2:**
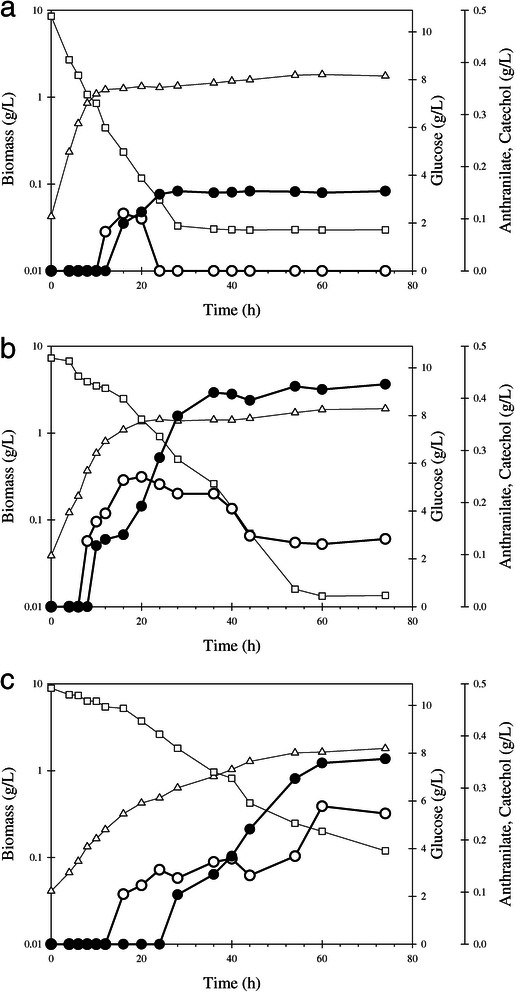
**Flask cultures of** ***E. coli*** **W3110** ***trpD9923*** **derivative strains** ***.*** **a)** *9923*/pTrc-*ant3,* **b)** *9923*/pJLB*aroG* ^fbr^ *tktA*/pTrc-*ant3* and **c)** *9923 PTS* ^*−*^/pTrc-*ant3.* Open triangles; biomass (g/L), open squares; glucose (g/L), open circles; anthranilate (g/L), closed circles; catechol (g/L).

To determine if expression of AntDO in these strains would cause the transformation of anthranilate to catechol, they were characterized in shake flask cultures using mineral medium supplemented with 10 g/L glucose. (Figure 2 and Table 2). The strains W3110 *trpD9923*/pTrc-*ant3* and W3110 *trpD9923*/pJLB*aroG*^fbr^*tktA*/pTrc-*ant3* showed similar μ values, whereas *q*_Glc_ was lower for the latter strain. As a result of PTS inactivation, strain W3110 *trpD9923* PTS^−^/pTrc-*ant3* showed the lowest μ and *q*_Glc_. Catechol was detected in cultures with all strains, where the *Y*_Cat/Glc_ values corresponded to 24, 27 and 13% of the theoretical maximum for strains W3110 *trpD9923* PTS^−^/pTrc-*ant3*, W3110 *trpD9923*/pJLB*aroG*^fbr^*tktA*/pTrc-*ant3* and W3110 *trpD9923*/pTrc-*ant3*, respectively. Anthranilate was also detected in cultures with strains W3110 *trpD9923*/pJLB*aroG*^fbr^*tktA*/pTrc-*ant3* and W3110 *trpD9923*/pTrc-*ant3*, indicating its incomplete conversion to catechol. Considering that W3110 *trpD9923*/pJLB*aroG*^fbr^*tktA*/pTrc-*ant3* showed the highest catechol production capacity, it was selected for further characterization in bioreactor cultures.

**Table 2:** **Production of catechol in flask cultures by** ***E. coli 9923*** **derivatives**

**Strain**	**Biomass (g**_**DCW**_ **/L)**	**μ (h** ^**−1**^ **)**	***q***_**Glc**_ **(g**_**Glc**_ **/g**_**DCW**_ **⋅h )**	***q***_**Ant**_ **(g**_**Ant**_ **/g**_**DCW**_ **⋅h)**	**Anthranilate (g/L)**	**Catechol (g/L)**	***q***_**Cat**_ **(g**_**Cat**_ **/g**_**DCW**_ **⋅h)**	**Y**_**Cat/Glc**_ **(g**_**Cat**_ **/g**_**Glc**_ **)**
W3110 *9923/* pTrc-*ant3*	1.69 ± 0.06	0.21 ± 0.01	0.83 ± 0.02	0.05 ± 0.01	0.00 ± 0.00	0.15 ± 0.00	0.05 ± 0.01	0.05 ± 0.00
W3110 *9923* PTS^−^/pTrc-*ant3*	1.58 ± 0.03	0.08 ± 0.00	0.21 ± 0.05	0.03 ± 0.01	0.22 ± 0.01	0.37 ± 0.05	0.03 ± 0.01	0.09 ± 0.01
W3110 *9923*/ pJLB*aroG* ^fbr^ *tkt*A/pTrc-*ant3*	1.83 ± 0.01	0.18 ± 0.02	0.39 ± 0.07	0.07 ± 0.02	0.15 ± 0.03	0.41 ± 0.03	0.07 ± 0.02	0.10 ± 0.01

Cultures were carried out in M9 mineral medium at 37°C.

### Effect of media composition on catechol production in fed-batch bioreactor cultures

**Figure 3:**
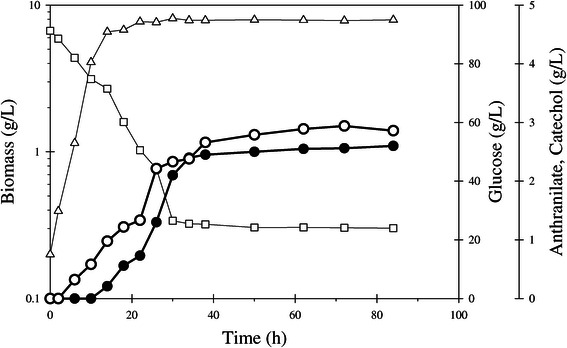
**Batch culture of strain W3110** ***trpD9923*** **/pJLB** ***aroG*** ^**fbr**^ ***tktA*** **/pTrc-** ***ant3*** **.** Mineral medium supplemented with 90 g/L of glucose and 30 g/L of yeast extract. Incubation temperature: 37°C. Open triangles; biomass (g/L), open squares; glucose (g/L), open circles; anthranilate (g/L), closed circles; catechol (g/L).

Strain W3110 *trpD9923*/pJLB*aroG*^fbr^*tktA/*pTrc-*ant3* was evaluated in fed batch cultures using M9 medium supplemented with 90 g/L of total fed glucose and yeast extract 30 g/L at 37°C. These culture conditions were chosen since they enabled the production of 14 g/L of anthranilate by strain W3110 *trpD9923*/pJLB*aroG*^fbr^*tktA* [8]. Strain W3110 *trpD9923*/pJLB*aroG*^fbr^*tktA*/pTrc-*ant3* displayed a μ of 0.17 ± 0.03 h^−1^ and a maximum biomass concentration of 10.44 ± 1.14 g/L at 30 h of culture (Figure 3, Table 3). Under these conditions, this strain produced 2.51 ± 0.21 g/L of catechol and 2.86 ± 0.09 g/L of anthranilate.

**Table 3:** **Production of catechol in bioreactor cultures by** ***E. coli 9923*** **derivatives**

**Culture conditions/Strain**	**Biomass (g**_**DCW**_ **/L)**	**μ (h** ^**−1**^ **)**	***q***_**Glc**_ **(g**_**Glc**_ **/g**_**DCW**_ **⋅h)**	***q***_**Ant**_ **(g**_**Ant**_ **/g**_**DCW**_ **⋅h)**	***q***_**Cat**_ **(g**_**Cat**_ **/g**_**DCW**_ **⋅h)**	**Anthranilate (g/L)**	**Catechol (g/L)**	**Y**_**Ant/Glc**_ **(g**_**Ant**_ **/g**_**Glc**_ **)**	**Y**_**Cat/Glc**_ **(g**_**Cat**_ **/g**_**Glc**_ **)**
37°C, M9 +									
30 g/L Yeast Extract									
W3110*9923*/pJLB*aroG* ^fbr^ *tkt*A*	18.54 ± 0.19	0.18 ± 0.01	0.14 ± 0.05	0.02 ± 0.01	-	14.00 ± 0.07	-	0.20 ± 0.00	-
W3110 *9923*/pJLB*aroG* ^fbr^ *tkt*A/pTrc-*ant3*	10.44 ± 1.14	0.17 ± 0.03	0.13 ± 0.02	0.02 ± 0.00	0.02 ± 0.01	2.86 ± 0.09	2.51 ± 0.21	0.09 ± 0.02	0. 07 ± 0.02
37°C, M9									
W3110 *9923*/pJLB*aroG* ^fbr^ *tkt*A	7.66 ± 0.64	0.17 ± 0.01	0.12 ± 0.03	0.02 ± 0.00	-	8.18 ± 0.08	-	0.17 ± 0.01	-
W3110 *9923*/pJLB*aroG* ^fbr^ *tkt*A/pTrc-*ant3*	7.13 ± 0.37	0.16 ± 0.02	0.11 ± 0.01	0.02 ± 0.00	0.02 ± 0.00	2.12 ± 0.08	2.81 ± 0.15	0.09 ± 0.01	0. 08 ± 0.02
32°C, M9									
W3110 *9923*/pJLB*aroG* ^fbr^ *tkt*A	7.51 ± 0.44	0.14 ± 0.01	0.09 ± 0.01	0.02 ± 0.00	-	8.61 ± 0.06	-	0.18 ± 0.02	-
W3110 *9923*/pJLB*aroG* ^fbr^ *tkt*A/pTrc-*ant3*	6.92 ± 0.27	0.13 ± 0.01	0.07 ± 0.02	0.02 ± 0.00	0.02 ± 0.01	2.89 ± 0.15	4.47 ± 0.16	0.10 ± 0.02	0. 16 ± 0.02
28°C, M9									
W3110 *9923*/pJLB*aroG* ^fbr^ *tkt*A	7.37 ± 0.77	0.10 ± 0.01	0.08 ± 0.01	0.01 ± 0.00	-	6.28 ± 0.10	-	0.11 ± 0.01	-
W3110 *9923*/pJLB*aroG* ^fbr^ *tkt*A/pTrc-*ant3*	6.75 ± 0.34	0.12 ± 0.01	0.06 ± 0.00	0.01 ± 0.00	0.01 ± 0.01	2.21 ± 0.14	1.83 ± 0.13	0.09 ± 0.02	0. 06 ± 0.01

*Results from Balderas et al. [8].

Aromatic amino acids present in yeast extract are known to participate in transcriptional repression of genes from the common aromatic and tryptophan biosynthetic pathways, as well as having an inhibitory effect on enzymes from these pathways [18]. To determine if yeast extract has a negative impact on anthranilate and catechol synthesis capacity, further characterization of W3110 *trpD9923*/pJLB*aroG*^fbr^*tktA*/pTrc-*ant3* was performed using mineral M9 medium supplemented with 90 g/L of total fed glucose but lacking yeast extract. To determine anthranilate production under these culture conditions, strain W3110 *trpD9923*/pJLB*aroG*^fbr^*tktA* was also included in these experiments**.** Strains W3110 *trpD9923*/pJLB*aroG*^fbr^*tktA* and W3110 *trpD9923*/pJLB*aroG*^fbr^*tktA*/pTrc-*ant3* displayed lower final biomass concentrations when compared with cultures supplemented with yeast extract. Under these growth conditions the final concentration of anthranilate produced by strain W3110 *trpD9923*/pJLB*aroG*^fbr^*tktA* was 8.18 ± 0.08 g/L with a *Y*_Ant/Glc_ of 0.17 ± 0.08 g_Ant_/g_Glc_ (Table 3). Strain W3110 *trpD9923*/pJLB*aroG*^fbr^*tktA*/pTrc-*ant3* accumulated 2.81 ± 0.15 g/L of catechol and 2.12 ± 0.08 g/L of anthranilate, with a *Y*_Cat/Glc_ corresponding to 21% of the theoretical maximum.

### Effect of incubation temperature on catechol production

The incubation temperature is a parameter that can have an important effect on the production level and activity of heterologous proteins expressed in *E. coli*, as well as the general cell physiology [19]. Therefore, to determine the effect of incubation temperature on catechol production, bioreactor cultures with strains W3110 *trpD9923*/pJLB*aroG*^fbr^*tktA* and W3110 *trpD9923*/pJLB*aroG*^fbr^*tktA*/pTrc-*ant3* using mineral medium supplemented with a total of 90 g/L of glucose were carried out at 37, 32 and 28°C.

Final biomass accumulation observed for strain W3110 *trpD9923*/pJLB*aroG*^fbr^*tktA* was unaffected at the different evaluated temperatures (Table 3). Anthranilate production and *Y*_Ant/Glc_ from cultures done at 32°C showed a slight increment when compared to experiments at 37°C, whereas at 28°C these parameters displayed a lower value (Table 3).

Growth and catechol production profiles for strain W3110 *trpD9923*/pJLB*aroG*^fbr^*tktA*/pTrc-*ant3* were differentially affected by the incubation temperatures employed. A reduction in *q*_Glc_ was evident at 32 and 28°C. The final catechol concentration in cultures at 28°C was the lowest observed. At 32°C, 4.47 ± 0.16 g/L of catechol and 2.89 ± 0.15 g/L of anthranilate were accumulated. The *Y*_Cat/Glc_ was 0.16 ± 0.02 g_Cat_/g_Glc_, 2-fold higher than that observed in cultures incubated at 37°C and corresponding to 42% of the theoretical maximum (Figure 3, Table 3).

## Discussion

Several microbial species can produce catechol by transformation of various aromatic substrates [2,3]. However, a drawback of such approach is that petroleum constitutes the raw material for synthesizing the aromatic substrates employed in these production processes. For this reason, there is considerable interest in the search of alternatives for the sustainable total synthesis of catechol employing renewable carbon sources as raw material. Production of catechol from a renewable starting material has been achieved by engineering metabolism in *E. coli*. The transformation of intermediate DHS to catechol via protocatechuic acid results from blocking the common aromatic pathway and expressing heterologous DHS dehydratase and protocatechuic acid decarboxylase activities [20]. Further strain improvement by increasing carbon flow to the common aromatic pathway has led to the production of catechol with a high yield from glucose [6].

In the present study, an alternate route for catechol production was developed by the heterologous expression of genes encoding AntDO from *P. aeruginosa* in derivatives of *E. coli* W3110 *trpD9923*, a mutant that overproduces anthranilate. When transformed with plasmid pTrc-*ant3*, strain W3110 *trpD9923* produced 0.15 g/L of catechol from glucose, thus showing AntDO catalytic activity in *E. coli*. When genes *aroG*^fbr^ and *tktA* were overexpressed in addition to *antABC*, a 2.7-fold increase in the final catechol concentration was observed, as well as the accumulation of 0.15 g/L anthranilate. This result is explained considering the expected increase in carbon flow to the common aromatic pathway caused by higher level activities of DAHP synthase and transketolase as it has been previously reported [14,21]. The observed accumulation of anthranilate under these conditions indicates that AntDO activity is not sufficient for consuming this precursor at the rate it is being produced. This is not observed in cultures with strain W3110 *trpD9923*/pTrc-*ant3* since the rate of anthranilate production is lower, therefore, this substrate can be completely transformed to catechol by AntDO.

Inactivation of PTS in strain W3110 *trpD9923* has been reported to cause an increase in the anthranilate yield from glucose [8]. Strain W3110 *trpD9923* PTS^−^/pTrc-*ant3*, displayed a 2.5-fold increase in catechol titer when compared to PTS^+^ strain W3110 *trpD9923*/pTrc-*ant3*. This result can be explained considering that aromatics precursor PEP is not consumed during glucose import in this mutant [14,22]. Therefore, a higher carbon flux to the common aromatic pathway is expected. Further improvement in the production performance for this strain could result from overexpression of genes *aroG*^fbr^ and *tktA*. However, experiments with strain W3110 *trpD9923* PTS^−^ revealed a negative effect on anthranilate production parameters when this strain was transformed with plasmid pJLB*aroG*^fbr^*tktA* [8]. This result can be explained considering that the PTS^−^ strain cannot cope with the metabolic burden resulting from plasmid replication and the high level expression of genes *aroG*^fbr^ and *tktA*, since its capacity to consume glucose is lower, when compared to a PTS^+^ strain. It remains to be determined if fine-tuning of expression level of these genes could result in lower metabolic burden and increased production capacity in the PTS^−^ strain.

Lower catechol and anthranilate production levels were observed when comparing rich to minimal medium cultures. This result is consistent with allosteric inhibition by tryptophan exhibited by the enzyme anthranilate synthase [23]. Strain W3110 *trpD9923* is a tryptophan auxotroph, therefore, this amino acid must be supplemented when employing a minimal medium. In the cultures performed in this study, the concentration of supplemented tryptophan was 20 μg/mL. The results observed in these experiments indicate that such tryptophan concentration is sufficient for allowing growth of the *trpD* mutant but not high enough to inhibit activity of anthranilate synthase.

Culture temperature has been reported to have an important effect on the yield and activity of heterologous proteins expressed in *E. coli*. Low growth temperatures can result in improved protein folding and reduced protease expression in the host strain [24,25]. AntDO activity was indirectly detected as a result of catechol synthesis, however, the AntDO proteins expressed in the strains in this study could not be identified in protein electrophoresis experiments, likely because expression level is below the limit of visual detection (results not shown). Therefore, it is not possible to determine if growth at temperatures lower than 37°C had an impact on the level of AntDO. The observed results suggest a higher AntDO activity at 32°C when compared to other culture temperatures. However, different growth temperatures could also alter cellular carbon flux distribution, having a positive or negative impact on catechol production. Therefore, to ascertain the mechanism(s) involved in the observed effects of growth temperature on catechol production capacity, further physiological strain characterization and *in vitro* analysis of AntDO protein level and activity should be performed.

The mechanisms most frequently cited to explain the toxicity of catechol in *E. coli* cells are: (*i*) the generation of reactive oxygen species (ROS) by redox reactions; (*ii*) DNA oxidative damage; (*iii*) protein damage by sulphydryl arylation or oxidation; and (*iv*) interference with electron transport in energy transducing membranes [26]. A negative effect of catechol on *E. coli* growth and production capacity for the aromatic intermediate DHS has been demonstrated starting at 0.275 g/L and 2.75 g/L, respectively [7]. In the bioreactor cultures performed in this study, catechol reached final concentrations spanning a range of 1.83 to 4.47 g/L. Therefore, a toxic effect of catechol is expected under these conditions, this explains the lower observed μ when comparing cultures of anthranilate and catechol producer strains. This might also explain why the amount of catechol + anthranilate was lower when comparing cultures with strain W3110 *trpD9923*/pJLB*aroG*^fbr^*tktA* where only anthranilate was produced, with cultures of strains expressing AntDO. A solution to this issue could be the use of *in situ* catechol extraction methods by employing an anion-exchange resin, as it has been reported [7].

Important differences exist concerning strain characteristics and catechol production efficiency when comparing synthesis routes starting from either DHS or anthranilate. When considering the catechol synthesis route starting from DHS, the maximum theoretical yield from glucose is 0.686 g_Cat_/g_Glc_, Five enzyme catalyzed steps are required to transform DHS to anthranilate. In one of these steps, PEP is consumed for the synthesis of intermediate 5-enolpyruvylshikimate-3-phosphate. Considering this extra carbon atoms requirement, the maximum theoretical yield from glucose for catechol synthesis from anthranilate corresponds to 0.376 g_Cat_/g_Glc_. On the other hand, the inactivation of gene *aroE* encoding SHIK dehydrogenase to cause DHS accumulation results in a deficiency to synthesize the aromatic amino acids and vitamins [6]. This multiple auxotrophy is a potential problem when employing a minimal medium in a production process. However, this deficiency can be circumvented by supplementing the culture medium with the required aromatic compounds or the intermediate shikimate. In contrast, inactivation of *trpD* causes a tryptophan auxotrophy, therefore, this amino acid must be supplemented when culturing in minimal medium. Considering these important differences, it remains to be determined which of the two catechol-production routes, based on DHS or anthranilate as starting precursors, is more cost-effective under industrial production conditions.

A yield of catechol from glucose of 33% has been reported with strains having the production route starting from DHS [6]. In another report, the yield value was increased from 5 to 7% when resin-based extraction of catechol was performed [7]. In this work, the highest observed catechol yield from glucose was 16% with strain W3110 *trpD9923*/pJLB*aroG*^fbr^*tktA*/pTrc-*ant3* (Table 3). However, this strain also accumulated anthranilate with a 10% yield from glucose. Therefore, it would be possible to further improve strain performance by increasing AntDO activity to promote the complete transformation of anthranilate to catechol and to improve the production process if a catechol extraction method is applied to reduce toxicity effects.

A novel approach for synthesis of the chemical precursor muconic acid from simple carbon sources in *E. coli* has been recently described [27]. This method is based on the transformation of anthranilate to catechol and then to muconic acid as a result of expression of heterologous AntDO and catechol 1,2-dioxygenase activities. Pathway optimization led to a strain that accumulated 689 mg/L of anthranilate and further modification resulted in a strain that produced 389 mg/L of muconic acid under shake-flask culture conditions. Although catechol was produced from anthranilate as a precursor under the studied conditions, no catechol accumulation in the culture medium was detected since it was completely transformed to muconic acid [27]. Considering the common elements in the metabolic engineering strategies for catechol and muconic acid production, it might be possible to adapt some of the strains and culture conditions developed here, to improve a process for muconic acid biosynthesis.

The strains and culture conditions developed in this work constitute a starting point for the generation of production processes for the synthesis of catechol from glucose at grams scale. This knowledge can be useful for devising and improving production processes for useful compounds derived from catechol.
